# Follow-up efficacy of physical exercise interventions on fall incidence and fall risk in healthy older adults: a systematic review and meta-analysis

**DOI:** 10.1186/s40798-018-0170-z

**Published:** 2018-12-13

**Authors:** Azza Hamed, Sebastian Bohm, Falk Mersmann, Adamantios Arampatzis

**Affiliations:** 1 Department of Training and Movement Sciences, Humboldt-Universität zu Berlin, Philippstr. 13, Haus 11, 10115 Berlin, Germany; 2 Berlin School of Movement Science, Berlin, Germany; 3 Department of Biomechanics, Faculty of Physical Therapy, Cairo University, Cairo, Egypt

**Keywords:** Fall prevention, Physical training interventions, Older adults, Fall risk, Fall incidence, Postural and balance perturbations

## Abstract

**Background:**

The risk of falling and associated injuries increases with age. Therefore, the prevention of falls is a key priority in geriatrics and is particularly based on physical exercising, aiming to improve the age-related decline in motor performance, which is crucial in response to postural threats. Although the benefits and specifications of effective exercise programs have been well documented in pre-post design studies, that is during the treatment, the definitive retention and transfer of these fall-related exercise benefits to the daily life fall risk during follow-up periods remains largely unclear. Accordingly, this meta-analysis investigates the efficacy of exercise interventions on the follow-up risk of falling.

**Methods:**

A systematic database search was conducted. A study was considered eligible if it examined the number of falls (fall rate) and fallers (fall risk) of healthy older adults (≥ 65 years) during a follow-up period after participating in a randomized controlled physical exercise intervention. The pooled estimates of the fall rate and fall risk ratios were calculated using a random-effects meta-analysis. Furthermore, the methodological quality and the risk of bias were assessed.

**Results:**

Twenty-six studies with 31 different intervention groups were included (4739 participants). The number of falls was significantly (*p* <0.001) reduced by 32% (rate ratio 0.68, 95% confidence interval 0.58 to 0.80) and the number of fallers by 22% (risk ratio 0.78, 95% confidence interval 0.68 to 0.89) following exercising when compared with controls. Interventions that applied posture-challenging exercises showed the highest effects. The methodological quality score was acceptable (73 ± 11%) and risk of bias low.

**Conclusions:**

The present review and meta-analysis provide evidence that physical exercise interventions have the potential to significantly reduce fall rate and risk in healthy older adults. Posture-challenging exercises might be particularly considered when designing fall prevention interventions.

**Electronic supplementary material:**

The online version of this article (10.1186/s40798-018-0170-z) contains supplementary material, which is available to authorized users.

## Key points

Physical exercise clearly reduces the follow-up risk of falling*.*

Exercise interventions for fall prevention may include stability-challenging conditions and perturbations.

Specification of such exercises (alongside intensity) and understanding of their physiological underlying effect is needed to ensure and improve effective retention of fall-related exercise benefits in the post intervention follow-up.

## Background

Aging is associated with a reduction of the functional and physiological capacity of the musculoskeletal and central nervous systems, which significantly affects motor performance [1–4]. It is well evidenced that these age-related declines increase the incidence of falls and re-falls among older people [5], with one third of older adults above 65 years falling at least once a year [6, 7] and increasing fall rates in even older ages [8–10]. Falls in older adults occur mainly during dynamic daily tasks (e.g., walking and initiation of walking, and sitting down or lowering) and in the absence of external events [11–13], which indicates a reduced ability of effective internal control and execution of regular dynamic movements. In the face of external hazards that occur during daily life tasks, such impairments of motor responses lead to even higher risk of stability loss [14–17]. When postural/dynamic stability cannot be maintained and a fall event occurs, injury incidence is particularly high in older adults. Falls are one of the leading causes of injury-related hospital admissions in this age group [18] and are often followed by functional dependence, serious or fatal injuries, fractures, and high morbidity [19].

The decline in motor performance is caused by diverse age-related changes across the many different levels of the human organism, e.g., central nervous and musculoskeletal. Among others, muscle weakness with aging, so-called sarcopenia [20], is a key factor that determines stability control and recovery responses following sudden threats [21–25]. The loss of muscle mass, which occurs due to a reduced number of motor units and size of single muscle fibers, as well as a decrease of voluntary activation [26–30], leads to a decline of the muscle force capacity [1, 2, 28, 31, 32]. As degenerative effects predominantly affect fast twitch fibers [33] and muscle fascicle length decreases as well [34], the mechanical power (product of force and velocity) as a predictor of the muscle’s functional capacity during dynamic stability threats [35] is affected in a twofold manner [36, 37]. Consequently, studies [21, 22] have demonstrated deficits in the execution of fundamental stability control mechanisms (e.g., modulation of the base of support and counter segment rotations around the center of mass [38]) in older adults, which likely contributes to the limited ability to regain stability following sudden unstable conditions [22].

Current reviews and guidelines regarding the prevention of falls consistently recommend physical exercises [39–43] using strength, balance, mobility, and perturbation training paradigms [40, 43–45] to counteract the decline of motor performance. Moreover, Tai Chi contains balance-challenging slowly performed movements and has been recommended for fall prevention in older adults [46, 47]. In fact, the body of randomized controlled trials shows that training of this kind in healthy older adults has the potential to improve strength [48–52], mobility [48, 52], stability, and balance control [50, 52, 53] and reduce the risk of falling [54] and related injuries [40, 49, 55, 56], within and after the intervention period [52, 57, 58]. Previous meta-analyses allowed for conclusions on the most effective characteristics of exercise training interventions with respect to the reduction of the risk of falling [44, 59, 60]. However, these meta-analyses did not distinguish between studies that assessed the effects occurring during the intervention time and studies that assessed only the follow-up period, i.e., after finishing the treatment. Thus, the question of how much of these benefits of training persist over a longer time period and transfer to daily life after completion (i.e., follow-up effects) is still not fully understood. As motor learning and neuromuscular plasticity in older adults is largely preserved [61–67], older adults are capable of an improvement and long-term retention of effective stability control mechanisms as well as gains in functional capacities, both of which are necessary to compensate for challenging balance conditions [64, 68]. Therefore, it can be argued that exercise interventions may improve relevant key factors of successful reactive postural responses to sudden postural threats occurring during daily life. However, although there is broad evidence on acute fall-related benefits of exercise interventions (e.g., strength gains, stability control improvements) [40, 44, 49, 55, 56, 69], little is known about how fall prevention interventions actually translate into a reduction of falls in time periods after participation, i.e., retention or follow-up. Further, the small sample sizes and diverse exercise approaches compromise the conclusions drawn from single randomized controlled trials.

The scope of the current review is to provide an analysis of healthy older adults whose fall occurrences are not co-affected by an additional factor, i.e., a particular pathology. Some impairments may affect the physiological responsiveness to the training or would require adjusted exercise delivery strategies (e.g., group sizes and supervision). Consequently, to avoid a bias due to factors other than aging on the efficacy of physical exercise interventions for post intervention fall prevention, we included only healthy older adults in the present review and meta-analysis. Therefore, the purpose of the present review and meta-analysis was to investigate the efficacy of physical exercise interventions on post intervention fall prevention in healthy older adults (i.e., without neurological disease [e.g., Parkinson’s disease, stroke, or dementia/cognitive impairment], serious visual impairments [e.g., cataract, glaucoma, or color blindness], severe cardiac, pulmonary or musculoskeletal disorders, and severe osteoporosis, not living independently and not taking psychotropic drugs, that could influence fall outcomes). We searched for randomized controlled trials, examining the effect of different types and forms of physical exercises on fall rate (i.e., number of fall events) and fall risk (i.e., number of fallers) during the follow-up period. For the respective studies, we calculated the weighted average effect sizes and assessed the study quality and risk of bias.

## Methods

### Search strategy

Three electronic bibliographic databases (Web of Science, MEDLINE, and Scopus) were systematically searched (from inception till August 2018) using a combined set of terms related to physical exercises (interventions, exercises, exercising, training), older adult subjects (elderly, old, aged, age, senior, geriatric, aging, ageing) and falls (accidental fall, falling, slip, tripping) (see Additional file 1). Each term was mapped to MeSH (Medical Subject Headings) and controlled terms if available. Moreover, the reference lists of the eligible studies and of previous meta-analyses were screened for additional suitable titles.

### Study inclusion and exclusion criteria

**Fig. 1:**
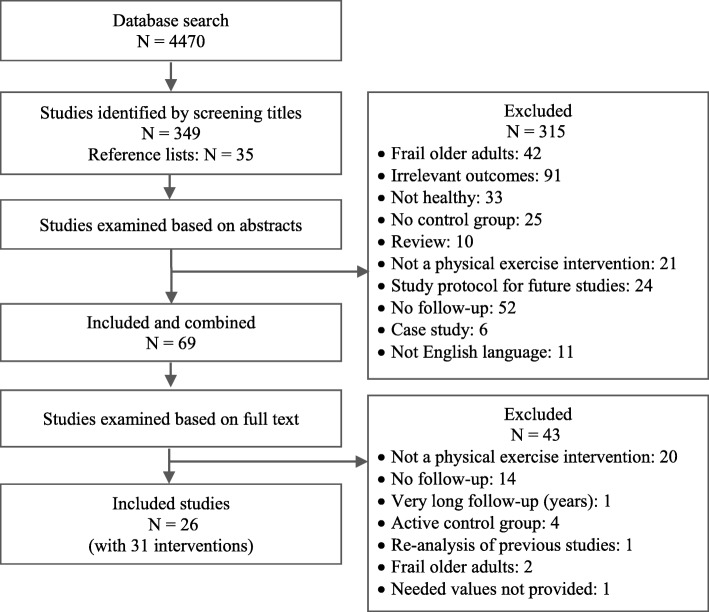
Flowchart of the systematic review process

The search results were evaluated at first by screening the study titles. Thereafter, abstracts and further the full texts were examined to determine their eligibility. A study was included when the following inclusion criteria were fulfilled: (a) investigation of fall incidence (fall rate) and/or number of fallers (fall risk) during (b) a follow-up period (started from the intervention’s end point) of at least 6 months after (c) a longitudinal (d) randomized controlled (level I) (e) physical exercise intervention (f) of at least 4 weeks on (g) healthy, (h) older adults (≥ 65 years). Studies which did not meet the inclusion criteria in this stage were excluded, and the respective exclusion reason was documented (Fig. 1). Finally, the reference lists of the eligible studies and of previous meta-analyses were screened for further articles. When a study presented different groups or intervention types and some of those did not meet the criteria, only the group or intervention that fulfilled the criteria was included. If an eligible study reported two or more interventions of different types of physical exercises, each intervention group was included separately. Note that studies with different follow-up durations and different exercise interventions were included, which might cause heterogeneity. The systematic review process of the present meta-analysis is presented in Fig. 1.

### Study quality assessment and risk of bias

We customized a methodological quality scale to assess the internal, statistical, and external validity of the included studies with regard to the concept of the present meta-analysis (Table 1). A positive point was assigned to each quality criterion if it was fulfilled. The quality score of each validity aspect (i.e., internal, statistical, and external) was expressed as the number of items with a positive score in percent of the total number of items. Thus, 100% indicates highest possible quality. The single section scores were then averaged to calculate the overall methodological quality of each study. However, a low result in the rating was not an exclusion criterion but allowed for an adequate interpretation of the single study outcomes in the context of the scope of the current meta-analysis. The calculation of the quality score of each validity section was done by counting the number of items with positive signs and dividing them by the total numbers of items to be expressed finally as a percentage. The single section scores were then averaged to calculate the overall methodological quality of each study.

**Table 1:** Criteria of methodological quality

Internal validity	Scoring
1. Study design	A positive point was assigned if the following aspects were considered: 1 Number of falls as an outcome measure 2 Number of fallers as an outcome measure 3 Healthy and not frail elderly 4 Follow-up period ≥ 6 months 5 Control group
2. Methods	A positive point was assigned if the following aspects were considered:
2.1 Quantification of fall incidence	A Criteria for the definition of a fall were provided and applied B Monthly returned fall diaries (i.e., fall calendar) C Reminder calls by the examiners to avoid forgetting reporting a fall [118–120] D Objective fall rate measurement as using sensor-based instruments (inertial sensors for daily life detection of falls) [121–124]
2.2 Intervention	A Physical form of exercise intervention B Group training under therapist supervision C Exercise material guidance for home training (only relevant for home training) D Controlling home visits by therapists for home training (only relevant for home training) E Duration of the intervention ≥ 4 weeks F At least two times per week [127, 128] G Session duration ≥ 15 min [127, 128] H Reporting compliance to the training (> 80%)
3. Cofactors	A positive point was assigned if the following aspects regarding the participants were considered: A Fall history in the previous 6 months or 1 year B Activity profile during follow-up C Influence of health status (diseases, medications) D Influence of cognitive ability
Statistical validity	Scoring
4. Statistical tests	A positive point was assigned if appropriate statistical tests were used
5. Power analysis	A positive point was assigned if the sample size was calculated based on an a priori power analysis
External validity	Scoring
6. Eligibility of sample and variables	A positive point was assigned if the intervention included as follows: 1 A representative sample 2 Appropriate report of the outcome variables
7. Description of the experimental protocol	A positive point was assigned if the following criteria were reported as follows: 1 Type of physical exercise intervention 2 Exercise descriptions and loading characteristics (e.g., intensity) 3 Intervention duration in weeks, training days per week, and session duration
8. Description of the participant sample	A positive point was assigned if the following criteria were reported as follows: A Age B Sex C Body height D Body mass E Activity level F Health status (medication) G Cognitive status H Fall history

Numbers indicate full-point items while letters indicate subcategories of a one full-point item

Note that the internal validity increases with using better methods for quantification of fall incidence and implementation of intervention

The risk of bias for each study was examined according to the Cochrane risk of bias tool [70] in which the following bias items were assessed: sequence generation, allocation concealment, blinding of participants and outcome assessors, incomplete outcome data, selective outcome reporting, and other sources of bias. The publication bias was tested by means of Egger’s test [71].

### Data extraction

The data of interest were extracted and organized in a table including all of the following information: authors’ names, participants’ characteristics (age, sex, and number), exercise protocol (type and description of exercises), intervention characteristics, and duration of follow-up period (for studies with more than one follow-up time point the latest one was chosen) as well as the main outcomes. If the outcome parameters (i.e., number of falls and number of fallers) were not reported in appropriate format (e.g., percentages, odds ratio, hazard ratio, or presented as a graph), the corresponding authors were contacted and asked to provide the missing values. Extracting the values visually from a graph was the last option. To avoid double inclusion of the same participants, one study [72] was excluded from the analysis as the data of the same participants were re-analyzed already in another included study [73] as stated by the authors.

### Statistical analysis

The fall rate (i.e., number of falls) and fall risk (i.e., number of fallers) were calculated from the completion time point of the intervention program until the end of the follow-up duration as a fall incidence rate ratio (value of intervention group divided by value of control group) and risk ratio for each study, respectively. The risk ratio was considered to account for the effect of multiple falls (more than one fall per person) [74] on fall rate ratio. Thus, a fall rate ratio and/or risk ratio below the value of one means lower risk in the intervention group than the untreated control group, while a value higher than one indicates a higher risk in the intervention group. The relative risk reduction was used to re-express the risk ratio and rate ratio as percentage reduction in number of fall events and number of fallers [75]. If the fall rate ratio or fall risk ratio were reported without the raw values of fall events and fallers [76–79], the ratios were taken directly from the respective study and the standard error was calculated from the 95% confidence intervals.

The single data were then pooled in a meta-analysis to estimate the effect sizes as weighted average overall fall rate and risk ratios, respectively. For this reason, a random-effects model of the generic inverse variance method was used because it gives more weight to the studies with small standard errors and takes into consideration the heterogeneity of the included studies [80, 81]. The presence of an overall effect of a physical exercise intervention on fall rate ratio and risk ratio during follow-up was tested accordingly [70]. The meta-analysis statistics and respective forest plots were performed using the software Review Manager (Version 5.2. Copenhagen: The Nordic Cochrane Centre, the Cochrane Collaboration, 2014)*.*

## Results

### Review statistics

A total number of 4470 studies were recorded after the database search (Fig. 1). The study titles were then checked for eligibility and at the same time, the duplicates were removed, yielding 349 potentially eligible studies. By reviewing the abstracts, the number of potentially eligible studies was 34. After reviewing the full text, 24 studies remained included. Screening of the reference lists of the included studies and of previous meta-analyses yielded an additional 35 related studies from which only two studies were eligible after checking the abstract and the full text. Finally, 26 studies were included in the current meta-analysis (Fig. 1). Three studies [76, 82, 83] reported two different intervention groups while one study reported three different intervention groups [51]. Each intervention group of these studies was included separately as a single study data set, increasing the total number of included interventions to 31. Thus, in the manuscript and analysis, we will henceforth refer to the 31 included interventions instead of the included studies.

### Description of the included studies

The present systematic review included in total 31 interventions (participants in total *n* = 4739), and their characteristics are summarized in Table 2. Twenty-four interventions reported both the fall rate and fall risk while three interventions investigated the fall risk only [50, 84, 85] and four interventions reported only the fall rate [83, 86, 87]. The mean age of the included participants was 74.1 ± 4.3 years. In the 26 interventions that reported the sex distribution of their participants, in total, 3240 were females and 735 males. The mean follow-up duration of all interventions was 12.43 ± 5.58 months. The types of the implemented physical exercises were combined balance and strength training (*n* = 16), balance-challenging mobility exercises in terms of trail-walking, complex obstacle negotiation exercises and multi-target stepping tasks (*n* = 6), Tai Chi (*n* = 4), balance training (*n* = 2), and strength training (*n* = 2). The type of physical exercises was not reported in one of the included interventions [85].

**Table 2:** Summary of the included interventions

No.	Study	Participants^a,b^	Exercise protocol	Intervention and follow-up	Outcome
1	Ballard et al. [90]	Total: *n* = 39 IG: *n* = 20, 72.4 ± 6.5 years, 20 F CG: *n* = 19, 73.4 ± 5.4 years, 19 F	Functional balance exercises (one leg balance tasks, half squats, lunges, and standing leg raises), strength exercises with elastic bands while seated (2 sets of 10 repetitions), low-impact aerobics routine (walking, stepping, and lunging in different directions while using intermittent arm motions)	*Intervention*: Duration: 3.8 months Session: 1 h Frequency: 3×/week Format: group Home exercise: no *Follow-up*: 12 months	No significant reduction in fall rate and risk
2	Beyer et al. [50]	Total: *n* = 53 IG: *n* = 24, 78.6 ± 5.1 years, 24 F CG: *n* = 29, 77.6 ± 4.4 years, 29 F	Standard resistance exercise (70–75% of 1 RM), balance training, and flexibility	*Intervention*: Duration: 6 months Session: 1 h Frequency: 2×/week Format: group Home exercise: no *Follow-up*: 12 months (starting from begin of study)	No significant reduction in fall risk
3	Fitzharris et al. [73]	Total: *n* = 272, 76.1 ± 5 years IG: *n* = 135 CG: *n* = 137	Strength and balance exercises supplemented with daily home exercises, 30–35% of the exercise contents were balance related	*Intervention*: Duration: 3.8 months Session: 1 h Frequency: 1×/week Format: group Home exercise: yes *Follow-up*: up to 18 months (starting from begin of study)	Significant reduction in fall rate and risk
4,5	Freiberger et al. [82]a,b	Total: *n* = 217 IG (a): *n* = 65 (62 analyzed), 76.4 ± 4.2 years, 31 F IG: (b): *n* = 69 (65 analyzed), 75.4 ± 3.8 years, 27 F CG: *n* = 83 (74 analyzed), 76.5 ± 3.9 years, 39 F	IG (a): strength (20%), balance (20%), motor coordination (30%), competence (15%), and perceptual training (15%) IG (b): strength and flexibility training (33%), balance and motor coordination training (33%), and endurance training (33%)	Intervention for (a) and (b): Duration: 4 months Session: 1 h Frequency: 2×/week Format: group Home exercise: yes *Follow-up*: 12 months	Significant reduction in fall rate and risk in IG (b)
6	Halvarsson et al. [94]	Total: *n* = 59 IG: *n* = 38 (30 analyzed), 76 years, 21 F CG: *n* = 21 (18 analyzed), 78 years, 15 F	Progressive balance training program that includes dual- and multi-task exercises (cognitive and/or motor)	*Intervention*: Duration: 3 months Session: 45 min Frequency: 3×/week Format: group Home exercise: no *Follow-up*: 15 months (starting from begin of study)	No significant reduction in fall rate and risk
7, 8	Iliffe et al. [83]a,b	Total: *n* = 572 IG: (a): *n* = 184, 72.9 years IG: (b): *n* = 178, 72.8 years CG: *n* = 210, 73.1 years	IG (a): Fall-management exercise program of progressive muscle strengthening, progressive balance retraining, bone loading, endurance (including walking) and flexibility training, functional floor skills, and adapted Tai Chi IG (b): Moderate intensity muscle strength, balance retraining, and walking plan	*Intervention*: Duration: 6 months Session: 1 h for group-based, 30 min for home-sessions Frequency: 3×/week Format: IG (a) home and group-based, IG (b) home-based only Home exercise: yes *Follow-up*: 18 months after the end of intervention	Significant reduction in fall rate
9	Kamide et al. [95]	Total: *n* = 57 IG: *n* = 28 (23 analyzed), 71.0 ± 3.8 years, 23 F CG: *n* = 29 (27 analyzed), 70.9 ± 3.4 years, 27 F	Thera-Band moderate intensity strength exercises for hip and knee, four exercises, 1–2 sets of 15 repetitions, balance training in terms of fast stepping exercises in AP and ML directions 10 repetitions in each direction for right and left leg, and impact training in form of heel drop and tip toes exercises, 60–100 repetitions	*Intervention*: Duration: 6 months Session: not stated Frequency: 3×/week Format: home-based Home exercise: yes *Follow-up*: 6 months after the end of intervention	No significant reduction in fall rate and risk
10,11,12	Karinkanta et al. [51]a,b,c	Total: *n* = 149 IG: (a): *n* = 37 (33 analyzed), 72.7 ± 2.5 years, 33 F IG: (b): *n* = 37 (31 analyzed), 72.9 ± 2.3 years, 31 F IG: (c): *n* = 38 (30 analyzed), 72.9 ± 2.2 years, 30 F CG: *n* = 37 (26 analyzed), 72 ± 2.1 years, 26 F	IG (a): Progressive resistance training with an intensity from 50 to 80% of 1 RM IG (b): Balance-jumping training: balance and agility training, jumps, modified and step aerobics, and impact exercises IG (c): Combined training: resistance and balance-jumping training in alternating weeks	*Intervention*: Duration: 12 months Session: 50 min Frequency: 3×/week Format: group Home exercise: no *Follow-up*: 12 months after the end of intervention	Significant reduction in fall rate and risk
13	Li et al. [89]	Total: *n* = 256, 77.48 ± 4.95 years, 179 F IG: *n* = 125 (95 analyzed) CG: *n* = 131 (93 analyzed)	Tai Chi Yang style	*Intervention*: Duration: 6.5 months Session: 1 h Frequency: 3×/week Format: group Home exercise: no *Follow-up*: 6 months	Significant reduction in fall rate and risk
14	Liu-Ambrose et al. [96]	Total: *n* = 59 IG: *n* = 31 (28 analyzed), 81.4 ± 6.2 years, 22 F CG: *n* = 28 (24 analyzed), 83.1 ± 6.3 years, 19 F	Otago exercise program of balance and strength retraining exercises	*Intervention*: Duration: 6 m Session: 30 min Frequency: 3×/week Format: home-based Home exercise: yes *Follow-up*: 6 months	Significant reduction in fall rate and risk
15	Logan et al. [129]	Total: *n* = 204, 78.86 years IG: *n* = 102 (82 analyzed), 67 F CG: *n* = 102 (75 analyzed), 65 F	Strength, balance exercises, and occupational functional therapy.	*Intervention*: Duration: 1.5 months Session: 2 h Frequency: 2×/week Format: group Home exercise: yes *Follow-up*: 12 m	Significant reduction in fall rate and risk
16	Logghe et al. [97]	Total: *n* = 269 IG: *n* = 138 (114 analyzed), 77.5 ± 4.7 years, 96 F CG: *n* = 131 (99 analyzed), 76.8 ± 4.6 years, 95 F	Tai Chi Yang style (10 positions)	*Intervention*: Duration: 3.2 months Session: 1 h Frequency: 2×/week Format: group Home exercise: yes *Follow-up*: 12 months	No significant reduction in fall rate and risk
17	Lord et al. [101]	Total: *n* = 197 IG: *n* = 100 (75 analyzed), 71.6 ± 5.5 years CG: *n* = 97 (94 analyzed), 71.7 ± 5.3 years	Strength exercises: lifting one’s own body weight (push up exercise), opposing muscle group resistive exercises, balance training: standing on the one leg, hand-eye and foot-eye coordination, ballgames requiring catching with the one hand while standing or moving, kicking a moving ball, throwing to a moving target, running under a skipping rope, and team ballgames	*Intervention*: Duration: 12 months (four 10–12 weeks terms) with 2 weeks interterm breaks and 5 weeks holiday break. Session:1 h Frequency: 2×/week Format: group Home exercise: no *Follow-up*: 12 months	No significant reduction in fall rate and risk
18	Means et al. [86]	Total: *n* = 99 IG: *n* = 47 (31 analyzed), 75 ± 4.9 years CG: *n* = 52 (34 analyzed), 75 ± 5.7 years	Balance and mobility exercises: postural control, flexibility, endurance walking, and muscle coordination exercises with training on obstacle courses	*Intervention*: Duration: 1.5 months Session: 1 h Frequency: 3×/week Format: group Home exercise: no *Follow-up*: 6 months	No significant reduction in fall rate
19	Means et al. [98]	Total: *n* = 338, 73.5 years, 193 F IG: *n* = 181 (144 analyzed) CG: *n* = 157 (94 analyzed)	Balance, strength, and mobility program: Active stretching, postural control, endurance walking, and coordination exercises to improve balance and mobility, strengthening exercises for abdomen, upper, and lower limb muscles	*Intervention*: Duration: 1.5 months Session: 90 min Frequency: 3×/week Format: group Home exercise: no *Follow-up*: 6 months	Significant reduction in fall rate and risk
20	Morgan et al. [84]	Total: *n* = 229 IG: *n* = 119, 81 ± 7.6 years, 86 F CG: *n* = 110, 80.1 ± 7.4 years, 76 F	Low-intensity exercise program in sitting and standing postures targeting muscle strength and joint flexibility	*Intervention*: Duration: 2 months Session: 45 min Frequency: 3×/week Format: group Home exercise: no *Follow-up*: 12 months	Significant reduction in fall risk in participants with low physical function level
21	Salminen et al. [130]	Total: *n* = 591 IG: *n* = 293 (290 analyzed), 251 F CG: *n* = 298 (292 analyzed), 246 F	Balance, coordination and weight shifting exercises, and circuit training for muscle strength	*Intervention*: Duration: 12 months Session: 45 min Frequency: 1×/2 weeks Format: group Home exercise: yes *Follow-up*: 24 months after the end of intervention	Significant reduction in fall rate and risk
22	Suzuki et al. [99]	Total: *n* = 52 IG: *n* = 28 (22 analyzed), 77.31 ± 3.40 years CG: *n* = 24 (22 analyzed), 78.64 ± 4.39 years	Muscle strength training, balance, and gait training, and Tai Chi exercises	*Intervention*: Duration: 6 months Session: 1 h Frequency: 1×/2 weeks Format: group Home exercise: yes *Follow-up*: 20 months	Significant reduction in fall rate and risk
23,24	Taylor et al. [76]a,b	Total: *n* = 684 IG (a): *n* = 233 (180 analyzed), 75.3 ± 7.0 years, 161 F IG (b): *n* = 220 (174 analyzed), 74.4 ± 6.2 years, 165 F CG: *n* = 231 (174 analyzed), 73.7 ± 6.2 years, 176 F	IG (a): Tai Chi exercises once weekly IG (b): Tai Chi exercises twice weekly	*Intervention*: Duration: 5 months Session: 1 h Frequency: 1×/week (IG a), 2×/week (IG b) Format: group Home exercise: no *Follow-up*: 17 months from study entry point	No significant reduction in fall rate and risk
25	Trombetti et al. [88]	Total: *n* = 134 IG: *n* = 66 (56 analyzed), 75 ± 8 years, 64 F CG: *n* = 68 (56 analyzed), 76 ± 6 years, 65 F	Music-based multi-task exercise program (i.e., Jaques-Dalcroze eurhythmics), e.g., handling of objects (balls), walking in time to the music, and responding to changes in the music’s rhythmic patterns. The exercises challenged the balance by requiring multidirectional weight shifting, walk-and-turn sequences, and exaggerated upper body movements during walking and standing	*Intervention*: Duration: 6.2 months Session: 1 h Frequency: 1×/week Format: group Home exercise: no Follow-up: 6 months	Significant reduction in fall rate and risk
26	Uusi-Rasi et al. [87]	Total: *n* = 175 IG: *n* = 86, 74.8 ± 2.9 years, 86 F CG: *n* = 89, 73.8 ± 3.1 years, 89 F	Progressive strength, balance, agility, and mobility training.	*Intervention*: Duration: 24 m Session: 1 h Frequency: 2×/week in the first year, 1×/week in the second year Format: group Home exercise: no *Follow-up*: 24 months after the end of intervention	No significant reduction in fall rate
27	Weerdesteyn et al. [100]	Total: *n* = 113 IG: *n* = 79 (78 analyzed), 73.4 ± 5.4 years, 63 F CG: *n* = 28 (28 analyzed), 74.9 ± 6.5 years, 19 F	Balance, gait, and coordination training in an obstacle course; e.g., walking over stepping stones. The second session in the week: walking with different speeds and directions. Practicing fall techniques in forward, backward, and lateral directions	*Intervention*: Duration: 1.2 months Session: 1.5 h Frequency: 2×/week Format: group Home exercise: no Follow-up: 7 months	Significant reduction in fall rate and risk
28	Whitehead et al. [85]	Total: *n* = 140 IG: *n* = 70 (58 analyzed), 79.5 ± 6.8 years, 48 F CG: *n* = 70 (65 analyzed), 76.1 ± 6.9 years, 52 F	No exercise descriptions are stated	*Intervention*: Duration: 3 months Session: 1–2 h Frequency: 1–2×/week Format: group Home exercise: no *Follow-up*: 6 months (from the moment of group assignment)	No significant reduction in fall risk
29	Yamada et al. [78]	Total: *n* = 60 IG: *n* = 30 (29 analyzed) CG: *n* = 30 (29 analyzed)	Trail-walking exercise: walking (multidirectional steps in the forward, backward, lateral, and oblique directions) from/around numbered flags. In addition to 20-min moderate intensity aerobic exercise, 20-min progressive strength training, 10-min flexibility and balance exercises	*Intervention*: Duration: 4 months Session: 1.5 h Frequency: 1×/week Format: group Home exercise: no *Follow-up*: 12 months	No significant reduction in fall rate and risk
30	Yamada et al. [79]	Total: *n* = 157 IG: *n* = 78 (72 analyzed), 85.8 ± 5.9 years, 63 F CG: *n* = 79 (73 analyzed), 85.3 ± 5.7 years, 64 F	Complex obstacle negotiation exercise; adding obstacles to the area of trail walk exercises and increasing the difficulty throughout the training	*Intervention*: Duration: 6 months Session: 45 min Frequency: 1×/week Format: group Home exercise: no *Follow-up*: 12 months	Significant reduction in fall rate and risk
31	Yamada et al. [77]	Total: *n* = 264 IG: *n* = 132 (112 analyzed), 76.2 ± 8.5 years, 67 F CG: *n* = 132 (118 analyzed), 77.2 ± 7.6 years, 65 F	Multi-target stepping tasks in the form of walking in different zigzag patterns, moderate intensity aerobic exercise (5 min), progressive strength training (10 min), flexibility, and balance exercises (15 min)	*Intervention*: Duration: 6 months Session: ~ 35 min Frequency: 2×/week Format: group Home exercise: no *Follow-up*: 12 months	Significant reduction in fall rate and risk

Unless otherwise indicated, the CG did not exercise. The studies followed by the letters a or b or c mean that they include different intervention groups, and each letter resembles one intervention group

*F* female, *IG* intervention group, *CG* control group, *AP* anteroposterior, *ML* medio-lateral, *RM* repetition maximum

^a^Age data are mean ± standard deviation

^b^The number in parentheses indicates is the number of the participants who continued the follow-up duration to the end, and their fall diaries were included in the final analysis

### Study quality assessment

The results of the methodological quality assessment of the included studies are presented in Table 3 and showed an achieved mean total score of 73 ± 11%, i.e., internal validity 81 ± 6%, statistical validity 67 ± 23%, and external validity 71 ± 14%, indicating acceptable methodological quality for most studies with regard to the scope of the present meta-analysis. The risk of bias assessment indicated a low risk of bias within studies (Table 4). However, the judgment of the allocation concealment and blinding of the assessor to the data domains was in some studies unclear since respective information was not reported (Table 4). The participants of the control group of six studies were physically active and performed low-intensity exercising such as aerobics and stretching exercises or simple indoor walking or balance and strength exercises [76, 78, 79, 86, 88, 89], and in two studies, the intervention program was continued partially during the follow-up period [89, 90] (Table 3). Both cases might have biased the intervention effect. Egger’s test for publication bias was not significant (*p* = 0.570), revealing low risk of publication bias.

**Table 3:** Methodological quality of the included studies

Study	Methodological quality																					
	Internal validity																					
	1.1^a^	1.2^a^	1.3^a^	1.4^a^	1.5^a^	2.1A^b^	2.1B^b^	2.1 C^b^	2.1D^b^	2.2A^b^	2.2B^b^	2.2C^b^	2.2D^b^	2.2E^b^	2.2F^b^	2.2G^b^	2.2H^b^	3A^b^	3B^b^	3C^b^	3D^b^	Score (%)
Ballard et al. [90]	+	+	+	+	+	–	–	–	–	+	+	/	/	+	+	+	–	+	–	+	–	79
Beyer et al. [50]	–	+	+	+	+	+	+	–	–	+	+	/	/	+	+	+	+	+	+	+	+	81
Fitzharris et al. [73]	+	+	+	+	+	+	+	–	–	+	+	/	/	+	–	+	–	–	–	–	–	77
Freiberger et al. [82]a,b	+	+	+	+	+	+	+	+	–	+	+	/	/	+	+	+	+	+	–	+	–	91
Halvarsson et al. [94]	+	+	+	+	+	+	–	–	–	+	+	/	/	+	+	+	–	+	–	+	+	82
Iliffe et al. [83]a,b	+	–	+	+	+	–	+	–	–	+	+	+	+	+	+	+	+	+	–	+	+	75
Kamide et al. [95]	+	+	+	+	+	+	–	–	–	+	/	–	–	+	+	+	+	+	–	+	–	81
Karinkanta et al. [51]a,b,c	+	+	+	+	+	–	–	–	–	+	+	/	/	+	+	+	–	–	–	+	–	76
Li et al. [89]	+	+	+	+	+	+	+	–	–	+	+	/	/	+	+	+	–	–	+	+	–	85
Liu-Ambrose et al. [96]	+	+	+	+	+	+	+	+	–	+	/	+	+	+	+	+	+	+	–	+	+	94
Logan et al. [129]	+	+	+	+	+	+	+	–	–	+	+	+	/	+	+	+	–	+	–	–	–	82
Logghe et al. [97]	+	+	+	+	+	–	+	–	–	+	+	/	/	+	+	+	–	–	–	–	–	76
Lord et al. [101]	+	+	+	+	+	+	+	–	–	+	+	/	/	+	+	+	–	–	–	+	–	82
Means et al. [86]	+	–	+	+	+	+	+	–	–	+	+	/	/	+	+	+	–	+	–	+	–	73
Means et al. [98]	+	+	+	+	+	+	+	–	–	+	+	/	/	+	+	+	–	–	–	+	–	83
Morgan et al. [84]	–	+	+	+	+	–	+	–	–	+	+	/	/	+	+	+	+	+	–	+	–	72
Salminen et al. [130]	+	+	+	+	+	+	+	–	–	+	+	+	+	+	–	+	–	+	–	–	–	83
Suzuki et al. [99]	+	+	+	+	+	–	+	–	–	+	+	+	–	+	–	+	+	+	+	–	–	83
Taylor et al. [76]a,b	+	+	+	+	+	+	+	+	–	+	+	/	/	+	+	+	+	+	–	–	+	91
Trombetti et al. [88]	+	+	+	+	+	+	+	–	–	+	+	/	/	+	–	+	–	+	–	+	–	83
Uusi-Rasi et al. [87]	+	–	+	+	+	+	+	–	–	+	+	/	/	+	+	+	–	–	–	+	+	73
Weerdesteyn et al. [100]	+	+	+	+	+	+	+	+	–	+	+	/	/	+	+	+	–	+	–	–	–	85
Whitehead et al. [85]	–	+	+	+	+	+	+	–	–	+	–	+	+	+	+	+	–	+	–	–	+	68
Yamada et al. [78]	+	+	+	+	+	+	+	–	–	+	+	/	/	+	–	+	–	–	–	–	+	80
Yamada et al. [79]	+	+	+	+	+	+	+	–	–	+	+	/	/	+	–	+	–	+	–	+	+	86
Yamada et al. [77]	+	+	+	+	+	+	+	–	–	+	+	/	/	+	+	+	–	+	–	+	+	89
Mean ± SD																						81 ± 6
Study	Methodological quality																					
	Statistical validity			External validity														Total score (%)				
	4^a^	5^a^	Score (%)	6.1^a^	6.2^a^	7.1^b^	7.2^b^	7.3^b^	8A^b^	8B^b^	8C^b^	8D^b^	8E^b^	8F^b^	8G^b^	8H^b^	Score (%)					
Ballard et al. [90]	+	+	100	+	+	+	+	+	+	+	–	+	+	+	–	+	94	91				
Beyer et al. [50]	+	+	100	+	+	+	+	+	+	+	+	+	+	+	+	+	100	94				
Fitzharris et al. [73]	+	+	100	–	+	+	+	+	–	–	–	–	–	–	–	+	53	77				
Freiberger et al. [82]a,b	+	–	50	–	+	+	–	+	+	+	–	–	+	+	–	+	57	66				
Halvarsson et al. [94]	+	+	100	+	+	+	+	+	+	+	–	+	+	+	+	+	72	85				
Iliffe et al. [83]a,b	+	+	100	+	+	+	+	+	+	+	–	+	+	+	+	+	72	82				
Kamide et al. 2009 [95]	+	–	50	–	+	+	+	+	+	+	+	+	+	+	–	+	72	68				
Karinkanta et al. [51]a,b,c	+	–	50	+	+	+	+	+	+	+	+	+	+	+	–	–	69	65				
Li et al. [89]	+	–	50	–	+	+	+	+	+	+	–	–	+	+	–	–	63	66				
Liu-Ambrose et al. [96]	+	+	100	+	+	+	+	+	+	+	+	+	+	+	+	+	75	90				
Logan et al. [129]	+	+	100	+	+	+	–	+	+	+	–	–	+	–	–	+	79	87				
Logghe et al. [97]	+	–	50	–	+	+	–	+	+	+	–	–	–	–	–	–	48	58				
Lord et al. [101]	+	–	50	–	+	+	+	+	+	+	–	–	+	+	–	–	63	65				
Means et al. et al. [86]	+	–	50	–	+	+	+	+	+	+	–	–	+	+	–	+	66	63				
Means et al. [98]	+	+	100	+	+	+	+	+	+	–	–	–	+	+	–	–	84	89				
Morgan et al. [84]	+	–	50	–	+	+	+	+	+	+	–	–	–	+	–	+	63	61				
Salminen et al. [130]	+	–	50	+	+	+	+	+	+	+	–	–	–	–	–	+	84	72				
Suzuki et al. [99]	+	–	50	–	+	+	+	+	+	+	–	–	+	–	–	+	63	65				
Taylor et al. 2012a,b [76]	+	–	50	+	+	+	–	+	+	+	–	–	–	–	+	+	79	73				
Trombetti et al. [88]a,b	+	–	50	+	+	+	+	+	+	+	–	+	+	+	–	+	94	76				
Uusi-Rasi et al. [87]	+	–	50	+	+	+	+	+	+	+	+	+	+	–	+	–	94	72				
Weerdesteyn et al. [100]	+	–	50	–	+	+	+	+	+	+	–	–	–	–	–	+	59	65				
Whitehead et al. [85]	+	–	50	–	+	–	–	+	+	+	–	–	–	–	+	+	46	55				
Yamada et al. [78]	+	–	50	–	+	+	+	+	+	–	–	–	–	–	+	–	56	62				
Yamada et al. [79]	+	–	50	–	+	+	+	+	+	+	+	+	–	+	+	+	72	69				
Yamada et al. [77]	+	+	100	+	+	+	+	+	+	+	+	–	–	+	+	+	94	94				
Mean ± SD			67 ± 23														71 ± 14	73 ± 11				

*Methodological quality: 1 Study design* | 1.1 Number of falls | 1.2 Number of fallers | 1.3 Healthy older adults | 1.4 Follow-up ≥ 6 months | 1.5 Control group **|** *2 Methods | 2.1 Fall Incidence |* 2.1 A fall definition | 2.1B Monthly diary |2.1C Reminder Calls| 2.1D Objective fall measure | *2.2 Intervention* | 2.2A Physical exercises | 2.2B Group training under therapist supervision | 2.2C Exercise material for home training | 2.2D Controlling home visits by therapists | 2.2E Intervention duration ≥ 4 weeks | 2.2F At least twice weekly | 2.2G Session duration ≥ 15 min | 2.2H Reporting compliance **|** *3 Cofactors* | 3A Previous fall history | 3B Reporting no exercise continuation during follow-up period | 3C Health status | 3D Cognitive status | *4 Appropriate statistical tests used* | *5 Power analysis* | *6 Eligibility* | 6.1 Appropriate and representative participant sample | 6.2 Appropriate representation of the outcome variables | *7 Description experimental protocol* | 7.1 Type of physical Intervention | 7.2 Exercise description | 7.3 Intervention duration in weeks, days and session time | *8 Description of the participant sample* | 8A Age | 8B Sex | 8C Body height | 8D Body mass| 8E Activity level | 8F Health status | 8G Cognitive status | 8H Fall history. The single criteria were rated (“+” = point, “−” = no point, “/” = not included) and used to calculate the quality score for each category (i.e., internal, statistical, and external validity). The average of the three scores gives the total score. ^a^A full point was assigned to each sub-category for the calculation of the score in the respective validity section ((assigned points/possible points)*100). ^b^The subcategories of the respective block were pooled to a single point (assigned points/possible points). The studies followed by the letters a or b or c mean that they include different intervention groups, and each letter resembles one intervention group

**Table 4:** Risk of bias assessment of the included studies according to Cochrane risk of bias assessment tool [75]

Study	Risk of bias						
	Sequence	Allocation	Blinding	Outcome	Report	Other	Notes
Ballard et al. [90]	Yes	Unclear	Unclear	Yes	Yes	Yes	Control group attended the exercise program in the first 2 weeks as a motivation. Examiners were not blinded to groups. Fall diaries were completed at 1-year follow-up, not on a monthly basis.
Beyer et al. [50]	Yes	Yes	Unclear	Yes	Yes	Yes	The follow-up started from the point of group assignment.
Fitzharris et al. [73]	Yes	Unclear	Unclear	Yes	Unclear	Unclear	
Freiberger et al. [82]a,b	Yes	Yes	Yes	Yes	Yes	Unclear	
Halvarsson et al. [94]	Yes	Yes	Unclear	Yes	Yes	Yes	Seventeen out of the 59 total had neurological and cardiovascular diseases. Fall frequency was assessed retrospectively at the end of the follow-up, not on a monthly basis calendars.
Iliffe et al. [83]a,b	Yes	Unclear	Yes	Yes	Yes	Yes	
Kamide et al. [95]	Yes	Yes	Yes	Yes	Yes	Yes	
Karinkanta et al. [51]a,b,c	Yes	Unclear	Unclear	Yes	Yes	Unclear	
Li et al. [89]	Yes	Unclear	Unclear	Yes	Yes	Unclear	
Liu-Ambrose et al. [96]	Yes	Yes	Yes	Yes	Yes	Unclear	
Logan et al. [129]	Yes	Yes	Yes	Yes	Yes	Yes	
Logghe et al. [97]	Yes	Unclear	Yes	Yes	Yes	Yes	
Lord et al. [101]	Yes	Unclear	Unclear	Yes	Yes	Unclear	
Means et al. [86]	Yes	Unclear	Unclear	Yes	Yes	Yes	Control group attended balance program without training on obstacle course.
Means et al. [98]	Yes	Yes	No	Yes	Yes	Yes	
Morgan et al. [84]	Yes	Unclear	Unclear	Yes	Yes	Unclear	
Salminen et al. [130]	Yes	Yes	Yes	Yes	Yes	Yes	
Suzuki et al. [99]	Yes	Unclear	Yes	Yes	Yes	Yes	
Taylor et al. [76]a,b	Yes	Yes	Yes	Yes	Yes	Unclear	The follow-up duration started from the entry point in the study to the final assessment point (i.e., the intervention duration is included in the follow-up period).
Trombetti et al. [88]	Yes	Yes	Yes	Yes	Yes	Unclear	The control group was a delayed intervention control group that started the same implemented intervention during the 6 months of follow-up.
Uusi-Rasi et al. [87]	Yes	Unclear	Yes	Yes	Unclear	Unclear	
Weerdesteyn et al. [100]	Yes	Unclear	Unclear	Yes	Yes	Unclear	Half of the intervention group was not randomly assigned. The follow-up started from the point of group assignment.
Whitehead et al. [85]	Yes	Yes	Yes	Yes	Yes	Unclear	The follow-up started from the point of group assignment.
Yamada et al. [78]	Yes	Yes	Yes	Yes	Yes	Unclear	
Yamada et al. [79]	Yes	Yes	Unclear	Yes	Yes	Unclear	
Yamada et al. [77]	Yes	Yes	Yes	Yes	Yes	Unclear	

*Sequence* Was the allocation sequence adequately generated? *Allocation* Was allocation adequately concealed? *Blinding* Was knowledge of the allocated intervention adequately prevented during the study? *Outcome* Were incomplete outcome data adequately addressed? *Report* Are reports of the study free of suggestion of selective outcome reporting? *Other* Was the study apparently free of other problems that could put it at high risk of bias? The studies followed by the letters a or b or c mean that they include different intervention groups, and each letter resembles one intervention group

### Meta-analysis of fall rate and fall risk

**Fig. 2:**
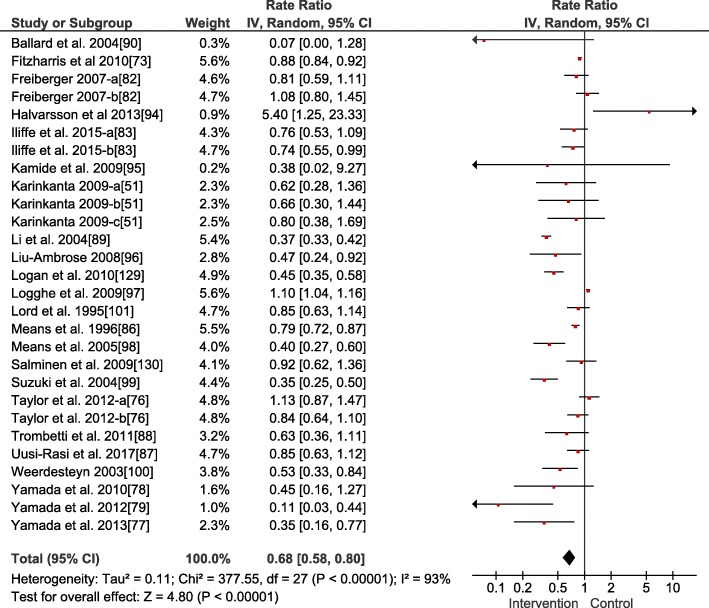
Forest plot for the meta-analysis of the fall rate (*n* = 4334). An inverse variance (IV) analysis was performed, and the 95% confidence interval (CI) is provided. The studies followed by the letters a or b or c mean that they include different intervention groups, and each letter resembles one intervention group

**Fig. 3:**
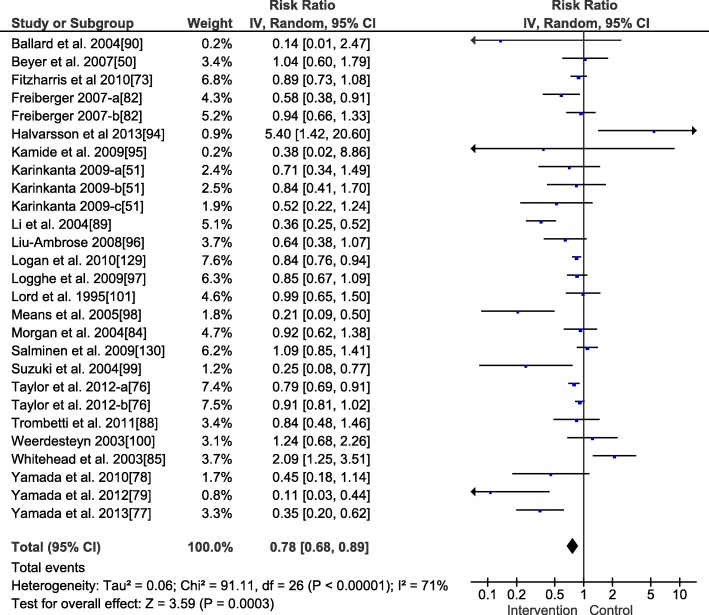
Forest plot for the meta-analysis of the fall risk (*n* = 3927). An inverse variance (IV) analysis was performed and the 95% confidence interval (CI) is provided. The studies followed by the letters a or b or c mean that they include different intervention groups, and each letter resembles one intervention group

The weighted average fall rate ratio (Fig. 2) of the included interventions was 0.68 (95% confidence interval 0.58, 0.80, *p* < 0.001, heterogeneity (*I*^2^) = 93%, *n* = 28) and the fall risk ratio 0.78 (95% confidence interval 0.68, 0.89, *p* < 0.001, *I*^2^ = 71%, *n* = 26, Fig. 3). Accordingly, relative risk reduction was 32% for the fall events and 22% for the number of older adults who fell, respectively. Studies with interventions focusing on stability-challenging conditions and/or perturbation-based exercises (i.e., performance of complex balance exercises and training of dynamic stability control in the context of uneven/unstable underfoot conditions) (*n* = 6) showed lower weighted average fall rates and risks of 0.52 for both (i.e., 48% reduction) compared to the interventions that focused on strength and balance combined (*n* = 16) with a fall rate ratio of 0.69 (i.e., 31% reduction) and a fall risk ratio of 0.79 (i.e., 21% reduction). Studies of Tai Chi interventions (*n* = 4) showed a fall rate ratio of 0.79 (i.e., 21% reduction) and a fall risk ratio of 0.72 (i.e., 28% reduction). Studies of interventions focusing on strength alone (*n* = 2) demonstrated a fall rate ratio of 0.62 (i.e., 38% reduction) and a fall risk ratio of 0.87 (i.e., 13% reduction). While studies of traditional balance intervention alone (*n* = 2) showed a fall rate ratio of 1.72 (i.e., no reduction) and a fall risk ratio of 1.92 (i.e., no reduction), balance functions were improved in these studies. However, a specific subgroup analysis on the type of the training was not conducted due to small subgroup sizes [70].

## Discussion

The current systematic review and meta-analysis assessed the follow-up efficacy of physical exercise interventions of different types on fall occurrences during daily life in healthy older adults. Twenty-six studies (31 interventions), with a total number of 4739 participants, were included giving a weighted average fall rate ratio of 0.68 and risk ratio of 0.78 (intervention/control) with low risk of publication bias. Thus, the analysis provides valuable evidence that physical exercise interventions have the potential to reduce the fall incidence and number of older adult fallers in the post intervention follow-up period by 32 and 22%, respectively.

In comparison, the training-induced reduction in fall incidence in the current meta-analysis was larger than those reported in the recent meta-analyses by Sherrington et al. [44], Gillespie et al. [40], Zhao et al. [49] and Sherrington et al. [59], i.e., 0.79 (21% reduction), 0.71 (29% reduction), 0.85 (15% reduction), and 0.83 (17% reduction), respectively. Also, the reduction in the number fallers was greater than those reported by Guirguis-Blake et al. [69] (risk ratio 0.89, 11% reduction), Tricco et al. [55] (0.83, 17% reduction), and Gillespie et al. [40] (0.85, 15% reduction). Additionally, the pronounced effect of exercise programs based on perturbation and stability training under challenging conditions on fall rate seen in the present meta-analysis (48% reduction) was larger than that reported in Sherrington et al. [44] (39%). To investigate the transfer and retention of training intervention effects on falls, the present meta-analysis included RCTs providing a follow-up time assessment after finishing the exercise intervention. However, the aforementioned meta-analyses [40, 44, 49, 55, 59, 60, 69] considered also studies in which the intervention time was part of the follow-up time (follow-up starts at intervention onset). From a physiological perspective, it can be expected that intervention benefits occur after a certain volume of training (number of sessions over time) [91–93] and might become functionally relevant (i.e., reduce falls) even later and, further, that biological responses progress over the time-course of intervention. Therefore, given that the period of intervention in this analysis may include a time when training effects have not (yet) become effective, and that this period might be a significant portion of the assessed overall follow-up time (e.g., in the present data set the average intervention time of 5.6 months would be almost one third of the overall follow-up time of 18.0 months), this might explain the lower observed effects on fall rate ratio and risk ratio reported in the previously published analyses compared with the current meta-analysis. Furthermore, for the same reason (follow-up time vs. follow-up time including intervention time), fewer and different studies were included in the present analysis compared to the previous meta-analysis (i.e. 10 [83, 88, 94–101] of the 26 studies included in the current meta-analysis were included in the 88 studies meta-analyzed by Sherrington et al. [44]).

The findings of the current analysis indicate that the reduction in the number of older persons who fell and the number of their fall events during daily life can be largely retained by about one third when participating in physical exercise interventions. Therefore, physical exercise interventions, being cheap and easy to implement (e.g., group settings in senior centers, home-based exercising), seem to be generally effective treatments of the age-related increase in fall risk. The implementation of such interventions may thus reduce fall-related injury clinical care burdens not only the individual but also the social health care systems.

The improvements in the general outcomes fall risk and number of fallers are likely the consequence of improvements in relevant intrinsic age-related fall risk factors. For example, balance and strength were seen to be improved after such intervention programs [50, 76, 88, 89, 94, 98, 100, 101] in association with improvements in more general physical functions (e.g., timed up and go test performance, functional reaching, and sit to stand time) [50, 77, 82, 101] and gait functional performance (e.g., gait velocity, stride length, and gait variability performance) [77, 88, 89, 100]. Accordingly, carryover effects were reported since the improvement in the gait and balance performance was retained after the cessation of the intervention program and during the follow-up duration [50, 88, 94]. However, improvements in these capacities and related functions might decrease or normalize over time when training is not continued, and persistent training is therefore needed to maintain the exercise-related benefits on fall risk factors.

In our analysis, the included studies applied a broad spectrum of physical exercises (balance, strength, mobility, combined balance and strength, Tai Chi, and balance-challenging mobility exercises), and except in a few cases, all these interventions decreased the risk of falling. With respect to efficacy, the interventions using stability-challenging conditions in their training and/or perturbation-based exercises (i.e., complex balance exercises and training of dynamic stability control in the context of uneven/unstable underfoot conditions) showed greater effects on fall rate and fall risk (i.e., 48% reduction for both) compared to interventions that focused on Tai Chi (21 and 28% reduction) and strength and balance combined (31 and 21% reduction). This may indicate a pronounced effectiveness of training interventions using stability-challenging conditions. However, it is important to note that this comparison was based on a very limited data set (*n* = 6, *n* = 4, and *n* = 16) and accordingly could not be statistically verified. Furthermore, not all of those studies were of appropriate methodological quality (Tables 3 and 4). Therefore, further systematic research is warranted to enable more definitive conclusions to be drawn. The indication of superior effects of perturbation-based training is nevertheless supported by the evidence from previous meta-analyses [44, 59, 60] and by current experimental studies that showed remarkable reductions in the annual self-reported fall risk of 43–50% following a single session of repeated unexpected slip exposures during walking [62, 102, 103]. Indeed, the degree of retention and transfer seem to depend on the intensity of the experienced perturbation, with greater effects seen with greater postural threats [104–107]. It might be argued that training balance control mechanisms using challenging conditions might improve the feedforward and feedback control of stability [68, 108–112] in an intensity-related manner, improving recovery performance following subsequent exposure to sudden perturbations during daily life situations. Moreover, it has recently been shown that specific strenuous balance exercises on unstable surfaces (challenging postural conditions) improved both recovery performance and muscle strength [113], thus increasing the efficiency of the intervention. The authors of the latter study suggested that the instability might increase muscle activation during exercising, which might stimulate strength gains alongside balance control mechanism improvements [113–115]. In this way, both deficient factors (balance and strength) could be trained at the same time. Therefore, including challenging balance conditions and perturbations may be a promising approach in fall prevention interventions.

The two studies which targeted balance alone [51, 53] showed no reductions in either fall rate (1.72) or risk ratio (1.92), although balance functions were improved in these studies. As reported by the authors [53], this was likely due to the training-related increases in activity level and self-confidence and a decreased fear of falling. Therefore, their exposure to balance-threatening events may have been increased. Again, due to the low number assigned to the different training components (stability-challenging perturbation training *n* = 6, combined strength and balance training *n* = 16, Tai Chi *n* = 4, traditional balance training *n* = 2, strength training *n* = 2), it was not possible to investigate any dose-response relationships in the current meta-analysis. However, it has been shown that intervention programs based on challenging balance exercises with a frequency of two and/or 3 h or more per week over a time period of 6 months have large effects on fall rate during and following the intervention program [44, 59, 60].

The total methodological quality score in the present meta-analysis ranged from 55 to 94%, with a mean of 73%, indicating moderate to high methodological quality of the included studies. However, several aspects were not present in every study. Fall rate was not investigated in three of the included interventions [50, 84, 85] while fall risk was not examined in four interventions [83, 86, 87]. Reporting fall incidence without reporting the number of single and multiple fallers (i.e., number of falls per patient) can bias the study results because certain participants may fall more often than others [74, 116]. Furthermore, an operational definition of a fall should be provided for seniors and health care providers to facilitate adequate quantification of falls [117]. The criteria of fall definition were not provided in nine of the included interventions [51, 83, 84, 90, 97, 99]. Furthermore, in order to reduce inaccuracies caused by memory lapses, it is recommended that fall diaries be completed on a daily basis and returned monthly rather than at the end of the follow-up period [118–120]. A validated instrument for detecting falls, e.g., using sensors [121–124] might reduce the aforementioned issues of self-reports. However, sensor-based wearable fall detection devices have been shown to be prone to errors such as false alarms and are not yet sufficiently precise and valid to be used in a scientific context [125, 126]. None of the included studies used such a methodology for the quantification of falls. The description of the experimental protocol and participants was appropriate in most of the included interventions, resulting in a moderate to high mean external validity score of 69%, although detailed information on the loading characteristics and detailed description of the exercise program were mostly missing. The risk of bias assessment indicated low risk for all interventions.

In the present systematic review and meta-analysis, only healthy older adults were included; thus, a generalization of the findings to older adults with different characteristics (e.g., frailty, diseases such as Parkinson disease) warrants confirmation.

## Conclusion

In conclusion, the present systematic review and meta-analysis provides evidence that physical exercise interventions significantly reduce fall rate and fall risk in healthy older adults during post intervention follow-up. This indicates that older adults benefit from physical exercise that targets age-related strength deficits and impaired stability control. However, detailed information on effective dose-response relationships remains sparse. Based on our results and other evidence, a possible recommendation could be to include challenging balance conditions and perturbations in exercise interventions to reduce the fall risk in older adults.

## Additional file

Additional file 1: Electronic bibliographic databases that were searched and applied respective search syntax. (DOCX 13 kb)

## Abbreviations

*I*^2^: Heterogeneity
MeSH: Medical Subject Headings
