# Supplementary material for: Follow-up efficacy of physical exercise interventions on fall incidence and fall risk in healthy older adults: a systematic review and meta-analysis
Source: Sports Med Open. 2018 Dec 13;4:56. doi: 10.1186/s40798-018-0170-z (PMC6292834; doi:10.1186/s40798-018-0170-z)
Supplement: Supplementary file 1 — Electronic bibliographic databases that were searched and applied respective search syntax. (DOCX 13 kb) [file 40798_2018_170_MOESM1_ESM.docx]

Additional file 1

| Web of science  (n= 2354) | TS: (intervention) OR TS: (training) OR TS: (exercises) AND TS: (falls) OR TS: (falling) OR TS: (tripping) OR TS: (slip) AND TS: (elderly) OR TS: (aged) OR TS: (ageing) OR TS: (aging) OR TS: (senior) OR TS: (geriatric) AND TS: (stability) OR TS: (balance) OR OR TS: (strengthening) OR TS: (gait) OR TS: (walk) OR TS: (walking) | 30.12.2017 |
| --- | --- | --- |
| PubMed  (n= 1692) | (aging[MeSH Terms]) OR age[Title/Abstract]) OR aged[MeSH Terms]) OR senior[Title/Abstract]) OR elderly[Title/Abstract]) OR geriatric[Title/Abstract]) AND accidental falls[MeSH Terms]) AND intervention[Title/Abstract]) OR exercising [MeSH Terms]) | 30.12.2017 |
| Scopus  (n= 424) | ( TITLE-ABS-KEY ( intervention )  OR  TITLE-ABS-KEY ( training )  OR  TITLE-ABS-KEY ( exercise ) )  AND  ( TITLE-ABS-KEY ( falls )  OR  TITLE-ABS-KEY ( falling )  OR  TITLE-ABS-KEY ( tripping )  OR  TITLE-ABS-KEY ( slip ) )  AND  ( TITLE-ABS-KEY ( elderly )  OR  TITLE-ABS-KEY ( aged )  OR  TITLE-ABS-KEY ( ageing )  OR  TITLE-ABS-KEY ( aging )  OR  TITLE-ABS-KEY ( senior )  OR  TITLE-ABS-KEY ( geriatric ) )  AND  ( TITLE-ABS-KEY ( stability )  OR  TITLE-ABS-KEY ( balance )  OR  TITLE-ABS-KEY ( strength )  OR  TITLE-ABS-KEY ( gait )  OR  TITLE-ABS-KEY ( walk )  OR  TITLE-ABS-KEY ( walking ) ) | 15.08.2018 |
